# Virucidal Activity of Plant Extracts against African Swine Fever Virus

**DOI:** 10.3390/pathogens10111357

**Published:** 2021-10-20

**Authors:** Małgorzata Juszkiewicz, Marek Walczak, Grzegorz Woźniakowski, Anna Szczotka-Bochniarz

**Affiliations:** 1 Department of Swine Diseases, National Veterinary Research Institute, Partyzantów 57 Avenue, 24-100 Puławy, Poland; marek.walczak@piwet.pulawy.pl (M.W.); grzegorz.wozniakowski@umk.pl (G.W.); anna.szczotka@piwet.pulawy.pl (A.S.-B.); 2 Department of Diagnostics and Clinical Sciences, Faculty of Biological and Veterinary Sciences, Nicolaus Copernicus University in Toruń, Lwowska 1 Street, 87-100 Toruń, Poland

**Keywords:** African swine fever, disinfection, plant extracts

## Abstract

African swine fever is one of the most dangerous and fatal swine diseases, described for the first time roughly a hundred years ago. Even now, there is neither a commercially approved vaccine nor treatment available. The only way to hinder further spread of the disease is by culling the affected herds and applying prevention based mainly on proper biosecurity. Due to growing awareness of the potential ASF threat among pig producers, disinfection processes are considered as one of the most important preventive measures. Currently, a variety of chemical compounds are applied for the disinfection of pig farms. Meanwhile, these chemicals may pose a potential risk, due to their toxic, irritant or corrosive effect. The aim of this study was to determine whether any plant-based natural compounds may show a virucidal effect against ASFV, and simultaneously be depleted of some of the side-effects typical for chemical compounds. Ideally, natural virucidal compounds should be safe for both humans and animals, biodegradable, easily available and inexpensive. Fourteen plant extracts were selected and screened for their virucidal effect against ASFV, using the suspension test inspired by the PN-EN 14675:2015 European Standard procedure. The results of our study showed that most of the tested plant extracts were ineffective against ASFV. Some extracts suspended in a hydroglycolic medium exhibited high virus titre reduction, but it was confirmed that the effect resulted from medium composition. However, a 1.05% peppermint extract showed high effectiveness against ASFV, reducing the virus titre by ≥4 log_10_, thus demonstrating that natural compounds used as virucidal agents could potentially be used in disinfection procedures, being both effective and harmless to humans and animals.

## 1. Introduction

**Figure 1:**
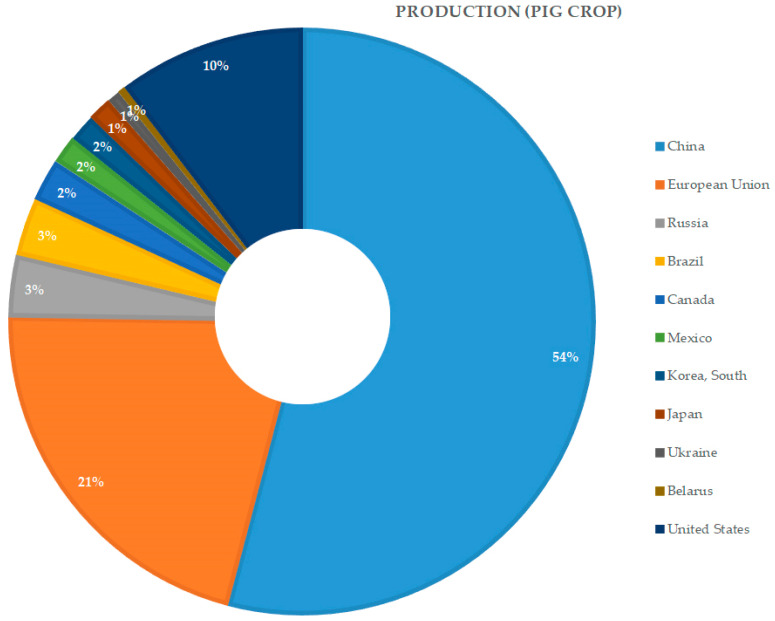
Global pork production in 2017 [8].

African swine fever (ASF), is one of the most serious diseases affecting domestic and wild representatives of the *Suidae* family (i.e., wild boar, warthogs) [1,2]. The disease is caused by the African swine fever virus (ASFV), a large DNA-virus that shows high genetic and antigenic diversity. At present, 24 genotypes and eight serogroups have been identified [3,4,5]. The antigenic diversity of ASFV and animals having no effective immune response are the main constraints in effective vaccine development [6]. The current epidemic began from a single introduction of the highly pathogenic ASFV genotype II to Georgia, in 2007. Since then, ASF has been spreading across Eastern and Central Europe, and in 2018 its devastating impact reached South-East Asia. Poland, as one of the leaders in pig production in Europe since 2014, is one of the most affected countries. Until now, in Poland—12,071 cases in wild boar and 469 ASF outbreaks in domestic pigs have been recorded, in total [7]. In 2018, the devastating impact of ASF reached China, accounting for approximately 50% of the world’s pork production, in 2017 [8] (Figure 1).

Since China accounts for approximately 50% of the world’s pork production, the emergence of ASF there caused a huge worldwide effect on pig production. Up until February of 2021, China’s government had confirmed 187 ASF outbreaks in 31 different administrative units [9,10,11]. The main reason for the high prevalence of ASFV in Chinese domestic pig herds has been explained as a lack of basic biosecurity measures among backyard and non-commercial pig holdings. Moreover, swill-feeding practices are still popular among non-commercial pig producers in China. These types of farms raise more than 60% of the pigs in China [5]. Until now, in Asia over 7,082,848 pigs have been culled due to ASF, which is equal to 82% of the total global reported losses [11,12].

Learning and understanding the ways of ASF introduction into the domestic pig population is crucial for prevention and intervention strategies. These measures present a single path to prevent further spread of the disease, especially in the absence of an effective vaccine or treatment [13]. The role of wild boar in ASF spread is pivotal, as they represent the main reservoir of the disease in the environment, mainly due to long ASFV persistence in wild boar carcasses [14,15]. ASFV may also replicate in soft ticks belonging to the *Ornithodoros* genus, present in Africa, making them actively involved in indirect ASFV transmission to the domestic and wild susceptible vertebrate hosts. Further studies have reported that a stable fly (*Stomoxys calcitrans*) may act as an ASFV mechanical vector, therefore it is recommended to apply mosquito nets at farm level [16]. It was later proved that only the ingestion of blood-fed ASFV contaminated flies by a susceptible pig may induce disease onset [17]. However, the weakest point in ASF transmission, from a contaminated environment to a pig holding, seems to be related to irresponsible human behavior. The main issue in ASF emergence in pig holdings is caused by neglecting basic biosecurity rules, including: inadequate protection of the farm against the entry of unauthorized people, no change of protective clothing, the presence of other domestic animals at the farm, the lack of disinfection procedures, swill-feeding or the application of disinfection agents with unconfirmed effectiveness. ASF is controlled by the culling of infected pigs and the implementation of high standards of biosecurity.

Global environmental change is affecting social health and threatens the future of many human beings. Contamination of the air, water and soil have led to the destruction of biodiversity that poses a risk to the ecosystems upon which the existence of all organisms depend. Environmental degradation, among others, is caused by overexploitation of its resources, overpopulation and contamination by detergents and chemicals [18]. The use of disinfectants, which has intensified recently, also has significant influence. In addition, disinfectants used by humans or in close proximity to animals can be dangerous to health and even life, after long-term exposure. For example, according to the U.S. Occupational Safety and Health Administration, formaldehyde should be handled as a potential carcinogen. It can cause asthma-like respiratory problems and skin irritation, such as dermatitis and itching, or after ingestion, even death [19,20]. This substantially limits the scope of its use as a disinfectant. This is not an isolated case, many chemical disinfectants cause more or less adverse health reactions, while having a detrimental effect on the environment. Therefore, the search for effective and safe antiviral agents has become an important area of study. The great diversity of plants and the lack of effective therapies and vaccines for ASF have urged a growing need for developing new, effective and safe agents to limit the spread of the disease. There are innumerable potentially useful plant extracts and herbs, and some of them have been shown to have great medicinal value in the prevention or treatment of viral diseases [21]. Scientific databases contain a huge number of articles on the antiviral, antifungal, antibacterial and even anthelmintic effects of medicinal herbs and plant extracts [22,23,24,25,26]. Moreover, several studies have shown that plants display antiviral activity both in vitro and in vivo [26]. However, their antiviral effectiveness may vary, depending on virus nucleoid acid type (RNA or DNA), virion architecture (enveloped or non-enveloped) and even against different strains of the same virus [25,27,28].

Plant extracts are usually derived from leaves, roots, fruits, stems, seeds, twigs, bark and flowers [28]. Selection of the plant part from which an extract should be prepared depends largely on the chemical composition of the plant. Extracts are obtained from dried, crushed vegetable material with solvents of different polarity. The most commonly used polar solvents are water, ethyl alcohol, glycerin and glycols, while non-polar are vegetable oils, isopropyl myristate and palmitate octyl. Choosing the proper raw material and solvent is pivotal for obtaining the most effective plant extract [29]. Medicinal plants contain primary and secondary substances in their composition. Primary metabolites are basic substances necessary for the life of every plant and fulfilling basic physiological functions (building, energy and spare). These include carbohydrates, fats, proteins, amino acids, enzymes and chlorophyll. Secondary metabolites, in turn, are products of the plant’s metabolism and usually are not crucial for basic life functions, they do not exist in all plants, but only in specific groups. Secondary metabolites include mainly saponins, coumarins, flavonoids, alkaloids, steroids, antibiotics, resins and lotions, essential oils, tannins, minerals and vitamins [30]. The effectiveness of some medicinal plants has been correlated with the presence of specific active substances. It has been proven that the main biologically active virucidal compounds of plants are terpenoids, alkaloids, stilbenes and flavonoids [31].

Due to the lack of information on the testing of plant extracts against ASFV [32,33], the aim of the present study was the determination of the antiviral activity of fourteen oil, hydroglycerin or hydroglycolic plant extracts (Table 1), using a method based on the PN-EN 14675:2015 European Standard.

## 2. Materials and Methods

### 2.1. Cells and Viruses

A Vero-adapted Ba71V strain was obtained from the African Swine Fever European Union Reference Laboratory (Valdeolmos, Madrid, Spain). A Vero cell line was obtained from ATCC (ATCC^®^ CCL-81TM) and subcultured in a Minimum Essential Medium (Gibco, Life Technologies, Carlsbad, CA, USA), supplemented with 10% fetal bovine serum (FBS, Gibco, Billings, MT, USA) and a 1% antibiotic-antimycotic solution (100×) (Sigma-Aldrich, St. Louis, MO, USA). The cultures were grown at 37 °C, in a humidified atmosphere of air containing 5% CO_2_.

### 2.2. Virus Stock Preparation

Sub confluent monolayers of Vero cells were infected with use of MOI ~0.01 and incubated at 37 °C, for 4–5 days until a 100% cytopathic effect was observed. In order to obtain [34] a sufficient virus titer (at least 10^6.5^ TCID_50_/mL), allowing for the demonstration of a 4 log titre reduction, after disinfectant treatment, viruses with a too low titer were subjected to three freeze/thaw cycles and precipitated, using the following buffer: 20% polyethylene glycol (PEG) and 2.5 M sodium chloride in a 2:3 buffer:virus ratio. The virus-buffer solution was agitated overnight at 4 °C, subsequently ASFV was pelleted by centrifugation at 13,000g for 90 min at 4 °C and resuspended in a 1/10 volume of the initial medium. The obtained virus stocks were titrated, aliquoted and stored at −80 °C. Virus titers were determined by 50% tissue culture infectious dose (TCID_50_/mL) titration, using the Spearman-Kärber method [35].

### 2.3. Plant Extracts

Four oil plant extracts were received from the Łukasiewicz Research Network—New Chemical Syntheses Institute (Pulawy, Poland) and ten extracts were provided by courtesy of the cosmetic company Bandi (Warsaw, Poland). Five of the 14 extracts were pure extracts, six were hydroglycerin extracts and the remaining three hydroglycolic extracts (Table 1).

Three selected concentrations of each plant extract were used. The maximum concentration that can be obtained by following the principles of the method is 80%. However, in this study, an intermediate concentration (60%) and a low concentration (30%) were also selected for analysis. All plant extracts were tested at the same three concentrations although, due to the medium being present in most of the extracts, the final concentration of eight of them was much lower; see Table 2. All selected concentrations of plant extracts were prepared immediately before use by dilution in hard water.

### 2.4. Diluents and Interfering Substances

All tested chemical compounds were diluted with water of standardized hardness, containing a defined concentration of Mg^+^, Ca^2+^, Cl^−^ and HCO^3−^ anions (pH 7). The hard water was prepared according to the PN-EN 14675:2015 European Standard. The suspension test was prepared with interference substances: BSA—bovine albumin 3.0 g/L (low level soiling) and BSA + YE—bovine albumin 10 g/L, plus a yeast extract 10 g/L (high level soiling) were prepared, according to the European standard PN-EN 14675:2015.

### 2.5. Test Conditions

Each extract concentration was tested in triplicate. One part of the virus suspension was mixed with one part of the interfering substances, respectively, with low level soiling, high level soiling and incubated at 10 ± 1 °C for 2 min ± 10 s. Subsequently, eight parts of the plant extract diluted to 1.25-fold of each tested concentration was added. The obtained mixture of the virus, tested extract and interfering substance was incubated at 10 ± 1 °C for 30 min ± 10 s. Afterwards, test tubes were placed on crushed ice (4 °C). Samples were immediately serially diluted (in quadruplicate) 10-fold (both the control virus and experimental virus suspensions) on a Vero cell culture, in 96-well plates. The plates were incubated for 7 days at 37 °C ± 2 °C, in air containing 5% CO_2_ and examined daily for the appearance of cytopathic effects (CPE). Finally, all plates were assessed for cytopathic effects by microscopic examination after 7 days post infection (dpi) A minimum 6.5 log_10_ (TCID_50_/_mL_) of virus titer in the control sample was required to demonstrate a ≥4 log reduction.

### 2.6. Cytotoxicity Reduction

Several plant extracts turned out to be cytotoxic to the Vero cells, therefore precluding in proper assessment of the test and demonstration of a 4 log_10_ titre reduction. Microspin S-400 HR columns (GE Healthcare, Fairfield, CT, USA) were used, in order to remove the cytotoxic extract from the samples, right after 30mins incubation of the tested and control samples.

### 2.7. Medium Antiviral Activity Assay

In order to assess the possible virucidal effect of the extract’s medium, a pure media (relevant for each extract) was prepared without an active substance and tested, according to Section 2.5 Test conditions. Final results are expressed as the logarithmic difference between the sample and control, and reduced additionally by the result of medium antiviral activity.

### 2.8. Test Controls

**Figure 2:**
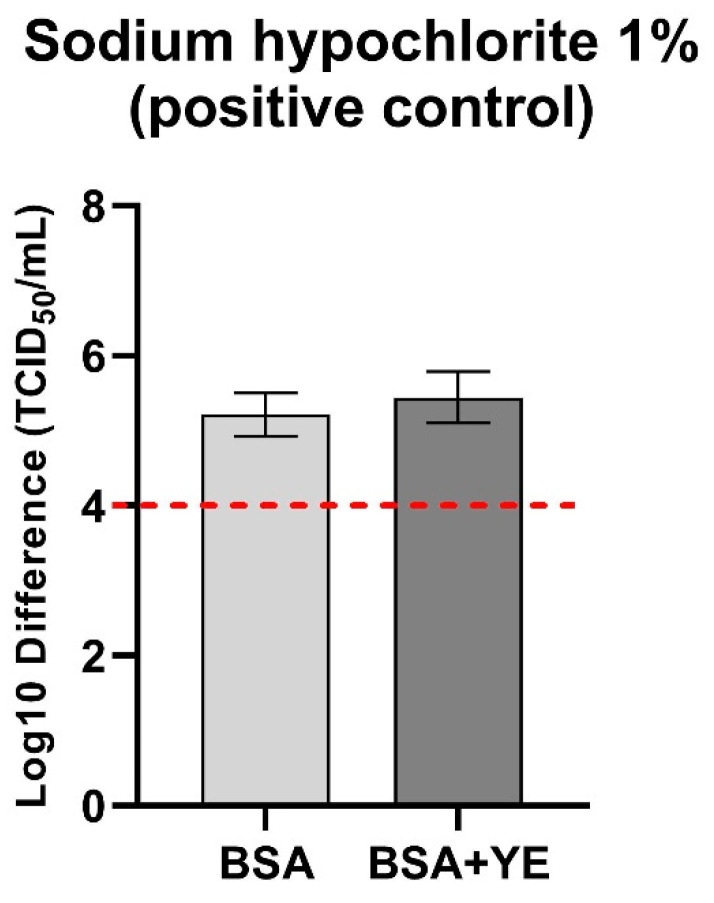
Mean difference in virus titer between positive control (sodium hypochlorite 1%) and virus control during study.

Both standard and cytotoxicity controls were processed in the same manner as the plant extract, which was replaced with hard water. In parallel, positive control for the virus susceptibility in virucidal assay was performed using 1% sodium hypochlorite. The test was valid when logarithmic reduction of virus titer caused by 1% sodium hypochlorite was ≥4 log_10_ in both soiling conditions (BSA and BSA + YE) (Figure 2).

### 2.9. Statistical Analysis

Statistical analyses were performed, using GraphPad Prism (version 8.4.3, GraphPad Software Inc., La Jolla, CA, USA). Analyses of the mean differences between each plant extract were shown with a standard deviation.

## 3. Results

**Figure 3:**
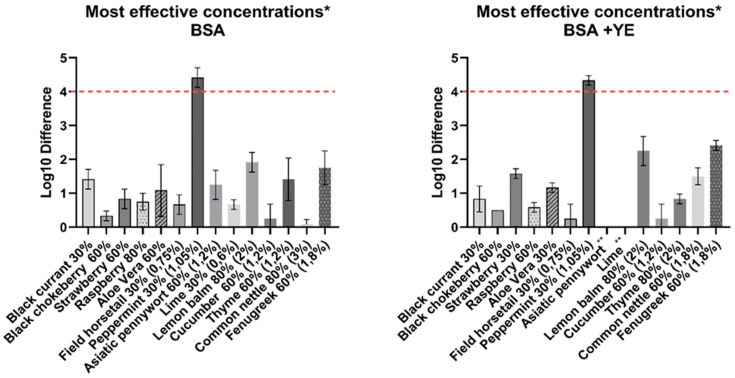
Most effective concentrations of tested plant extracts, in the presence of a low soiling level (BSA) and a high soiling level (BSA + YE). Maximum detectable log_10_ differences are presented. *—if the log difference was the same in one or more concentrations, the lower concentration was presented on the graph; **—no reduction in viral titer was observed.

The efficacy of the plant extracts was assessed by comparing the mean log reduction in the mixture of virus and tested plant extracts, with the logarithm of the virus control and the viral titer reduction value obtained by the medium. The extract was found to be effective when the difference between the control virus titre and the obtained titre in the sample was ≥4 log_10_. The collected results, after the necessary reductions, are included in Table 2. The most effective concentrations of the tested extracts are summarized in Figure 3.

**Figure 4:**
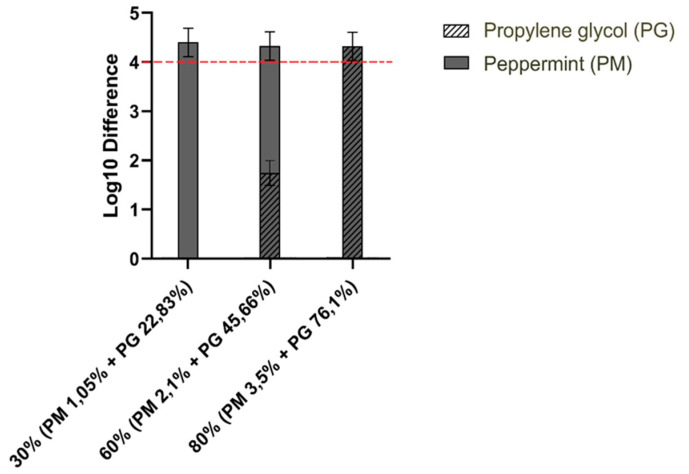
The final result of the antiviral efficacy of peppermint extract compared to the reduction achieved by the media.

Initially, three of fourteen extracts turned out to be effective against ASFV—peppermint, fenugreek and common nettle—showing >4 log_10_ reduction of viral titer (data not shown). The antiviral activity assay of the hydroglycolic medium showed that it was responsible for effectiveness at high (80%) and medium (60%) concentration, reducing the viral titre by >4 log_10_ and 1–2.4 logs_10_, respectively (Figure 4).

In addition, peppermint (3.5%, 2.1%) and fenugreek (3%, 1.8%) showed significant cytotoxicity. Both extracts were retested with the cytotoxicity assay. As a final result, fenugreek (3%, 1.8%), common nettle (3%, 1.8%) and peppermint (3.5%, 2.1%) were considered as ineffective. The hydroglycolic medium at 30% concentration did not cause virus inactivation. A similar result was obtained for the lowest concentration of the common nettle and fenugreek extracts. However, at the same level (30%) peppermint extract (1.05%) turned out to be effective against ASF, presenting 4.41(±0.23) log_10_ reduction (BSA) and 4.17 (±0.11) log_10_ reduction (BSA + YE), which corresponds to more than 99.99% pathogen reduction.

A relatively high reduction in virus titer of approximately 2log_10_ was observed for a 2% concentration of lemon balm. Interestingly, a higher efficiency was achieved under high level soiling conditions. Moderate effectiveness was demonstrated for 1.2% and 2% Thyme extract at the low soiling level—it reduced a virus titre by a maximum of 1.41(±0.51) log_10_ and 1.25(±0.40) log_10_, respectively. Despite the high initial concentration of the oil extracts (blackcurrant, black chokeberry, strawberry and raspberry), these demonstrated a low virucidal activity. Maximal observed viral titre reduction was observed for strawberry (30%)–1.58 (±0.11) log_10_, BSA+YE. Similarly, aloe vera extract was ineffective, causing maximal viral titer reduction of about 1log_10_ under both soiling conditions. Field horsetail, cucumber, asiatic pennywort and lime showed the lowest effectiveness. In the case of the last two extracts, the high soiling condition rendered them completely ineffective against ASFV.

**Figure 5:**
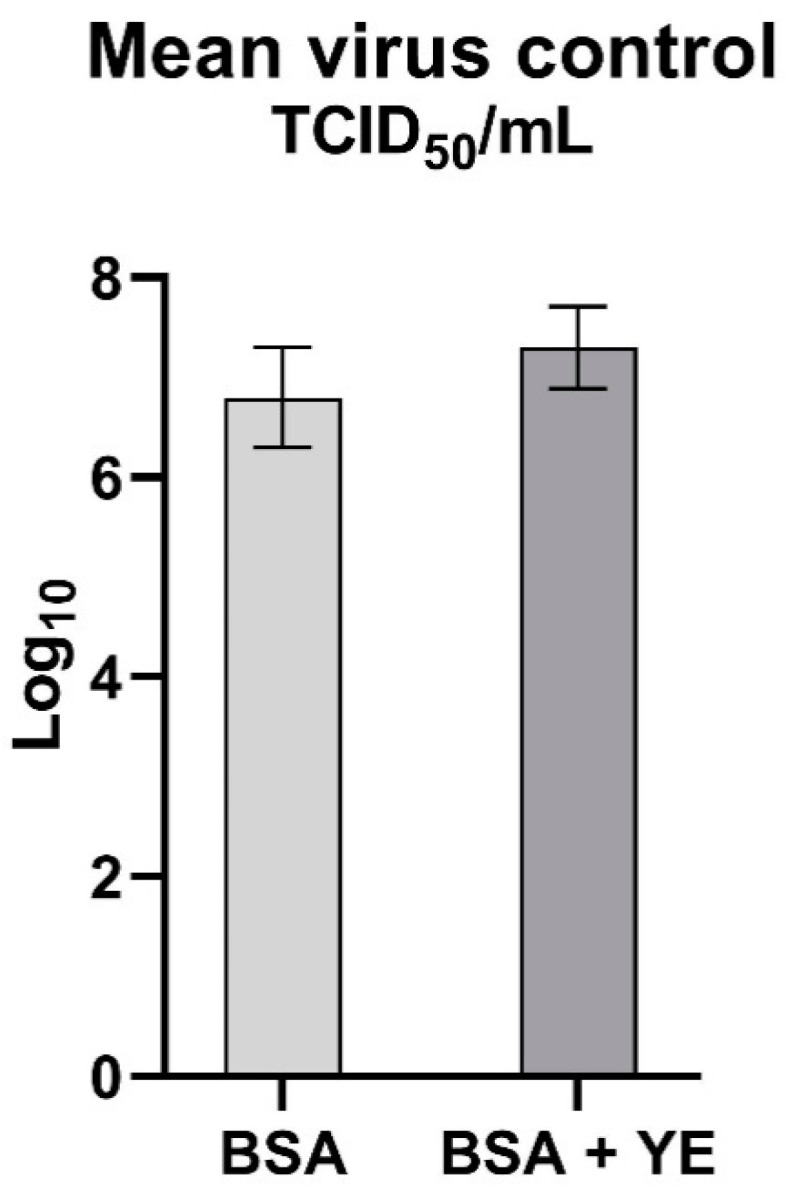
Mean titer of the virus control in two variants of soiling levels.

The mean virus titer value used in the study was 7.25 (±0.5) TCID50/mL for the high level soiling conditions, while 6.7 (±0.6) TCID_50_/mL for the low level soiling conditions (Figure 5) The difference confirmed the increased survivability of the viral population as the organic matter particles physically protect the virus from disinfectants and other antiviral agents, which may affect the assessment of the plant extract as a disinfectant [36]. Therefore, pre-cleaning cannot be ignored before disinfection. The effect of high-level soiling conditions on the effectiveness of plant extracts in most cases is also visible, in Figure 3.

## 4. Discussion

ASF is one of the most dangerous swine diseases that the world has faced during the last century, so far without an effective vaccine or treatment. The only solution to combat the disease is the culling of infected pigs or prevention consisting of compliance with the principles of biosecurity and effective disinfection. Due to the growing awareness of people related to ecology and the broadly understood care for the environment, much attention is paid to limiting the use of chemicals and focusing on the use of plants and their derivatives. Hence, medicinal products, cosmetics and detergents are often based on plant products.

Due to the lack of information on plant extracts and their virucidal effectiveness against ASFV [32,33], the aim of the present study, was to determine the virucidal activity of fourteen selected plant extracts (Table 1), based on the method inspired by the PN-EN 14675:2015 European Standard. Neither of the available studies have proven the effectiveness of the examined extracts. The lack of effectiveness arose not only from an insufficient reduction rate (0.7 log_10_), but it was also related to high cytotoxicity. According to the European Standard, the viral titre must be reduced by at least 4 log_10_ to consider a disinfectant effective.

This is the first study reporting the virucidal activity of plant extracts against ASFV by an in vitro suspension test, inspired by PN-EN 14675:2015. In this investigation, 14 plant extracts belonging to ten different plant families were tested. Despite many scientific studies confirming the virucidal effectiveness of blackcurrant, black chokeberry, strawberry and raspberry extracts [23,24,37,38,39], unfortunately we were unable to prove their effectiveness against ASFV. The preliminary tests of four oil extracts showed their very low solubility, which may have influenced the obtained results. Several of the tested plant extracts showed moderate virucidal activity, by decreasing the viral titre between 1–2 log_10_, however, they cannot be considered fully effective against ASFV, because they do not meet the 4 logs_10_ reduction criterion. Initially, fenugreek, common nettle and peppermint were found to be the most effective. However, after verifying the impact of hydroglycolic medium on its effectiveness, only peppermint showed confirmed virucidal efficacy by inactivating ASFV at 1.05% concentration, under low and high soiling conditions. It is noteworthy that propylene glycol (PG) (concentration ≥76.1%) was found to be effective (virus titer reduction ≥4 log10) against ASFV, and we were able to show its synergistic action in the case of peppermint. These results are partly supported by Kramer et al. where using acetone instead PG decreased hand disinfectant efficacy against other viruses [40]

Despite the fact that the virus is enveloped, and known to be susceptible to various disinfection strategies [41], only one of the fourteen tested extracts effectively inactivated the ASFV, which may indicate the moderate virucidal properties of the examined extracts, or a too low concentration was used. Peppermint virucidal efficacy with respect to enveloped viruses has already been proven in many previous studies [22,25,27,42] which support our results. On the basis of studies indicating the inactivating effect of menthol against herpes simplex virus infection (HSV-1 and HSV-2) [42] and analysis of the substances in the composition of the peppermint extract, it can be hypothesized that antiviral effectiveness against ASFV is related to the dominant amount of menthol in its composition (42.8%), which distinguishes it from the other two members of the Lamiaceae plant family. Other authors have shown that plant extracts from the Lamiaceae family were moderately effective, which is consistent with our results [43,44]. It can be assumed that their antiviral effectiveness is related to the presence of an unidentified, common component within these three plants, however, to confirm this hypothesis, more detailed research should be performed. Therefore, it can be concluded that thorough analysis of secondary substances in composition of plant extracts and testing them in higher concentrations is recommended in future studies.

In conclusion, our research showed that peppermint extract (1.05%) is virucidal against ASFV. In light of the obtained results, it can be concluded that the higher concentrations are also effective, albeit cytotoxic. The remaining thirteen plant extracts showed low or moderate virucidal activity against ASFV. High soiling was shown to have a significantly negative impact on disinfection effectiveness, which confirms the crucial role of pre-cleaning prior to proper disinfection.

Our research has proven the existence of a naturally-derived disinfectant, effective against the ASF virus, which may be safe for animals, humans and the environment, which is additionally ecological, biodegradable, inexpensive and easily available.

## Figures and Tables

**Table 1:** Tested extracts and their percentage composition.

Species (Family)	Common Name	Part Extracted	Extracts Ingredients
*Ribes nigrum* (Grossulariaceae)	Black currant	Seeds	*Ribes nigrum oil* extract-100%
*Aronia melanocarpa* (Rosaceae)	Black chokeberry	Seeds	*Aronia melanocarpa* oil extract-100%
*Fragaria ananasa* (Rosaceae)	Strawberry	Seeds	*Fragaria ananasa* oil extract-100%
*Rubus idaeus* (Rosaceae)	Raspberry	Seeds	*Rubus idaeus* oil extract-100%
*Thymus vulgaris* (Lamiaceae)	Thyme	Flower/Leaf	Glycerine-52.60% Water-45.0% *Thymus vulgaris* extract-2.00% Sodium benzoate-0.20% Potassium sorbate-0.20%
*Equisetum arvense* (Equisetaceae)	Field horsetail	Above ground parts	Glycerine-48.50% Water-48.50% *Equisetum arvense* extract-2.50% Sodium benzoate-0.25% Potassium sorbate-0.25%
*Mentha piperita* (Lamiaceae)	Peppermint	Leaf	Propylene glycol-76.1% Water-20.00% *Mentha piperita* extract-3.50% Sodium benzoate-0.20% Potassium sorbate-0.20%
*Aloe barbadensis* (Asphodelaceae)	Aloe Vera	Leaf	*Aloe barbadensis*-99.8% Sodium benzoate-0.1% Potassium sorbate-0.1%
*Centella asiatica* (Apiaceae)	Asiatic pennywort	Leaf	Glycerine-49.0% Water-48.50% *Centella asiatica* extract-2.00% Sodium benzoate-0.25% Potassium sorbate-0.25%
*Citrus aurantifolia* (Rutaceae)	Lime	Fruit	Glycerine-50% Water-47.50% *Citrus aurantifolia* extract-2.00% Sodium benzoate-0.25% Potassium sorbate-0.25%
*Melissa officinalis* (Lamiaceae)	Lemon balm	Leaf	Glycerine-50% Water-47.40% *Melissa officinalis* extract-2.00% Sodium benzoate-0.30% Potassium sorbate-0.30%
*Cucumis sativus* (Cucurbitaceae)	Cucumber	Fruit	Glycerine-50% Water-47.85% *Cucumis sativus* extract-1.75% Sodium benzoate-0.20% Potassium sorbate-0.20%
*Urtica dioica* (Urticaceae)	Common nettle	Leaf	Propylene glycol-79.0% Water-17.648% *Urtica dioica* extract-3.0% Phenoxyethanol-0.29% Methylparaben-0.062%
*Trigonella foenum-graecum* (Fabaceae)	Fenugreek	Seed	Propylene glycol-76.50% Water-20.00% *Trigonella foenum-graecum* extract-3.125% Phenoxyethanol-0.375%

Underline—the actual percentage of extracts in the tested solutions (excluding pure extracts without the medium).

**Table 2:** Logarithmic reduction of the ASFV titer in the presence of plant extracts. Contact time: 30 min. Temperature of incubation: 10 °C. Values were calculated as a mean of the 3 experiments, ±SD.

Plant Extracts	Tested Concentration of the Extract (Real Concentration of Active Compound)	Log_10_ Difference ** (±SD) (TCID_50_/_mL_)		Virucidal Effect (Reduction ≥ 4 Log_10_)	
		BSA	BSA + YE	BSA	BSA + YE
Black currant	80% (80%)	0.3 (±0.11)	0.1 (±0.1)	No	No
	60% (60%)	0.4 (±0.11)	0.0 (±0.0)	No	No
	30% (30%)	1.4 (±0.23)	0.8 (±0.31)	No	No
Black chokeberry	80% (80%)	0.33 (±0.11)	0.25 (±0.2)	No	No
	60% (60%)	0.33 (±0.11)	0.5 (±0.00)	No	No
	30% (30%)	0.08 (±0.11)	0.0 (±0.00)	No	No
Strawberry	80% (80%)	0.08 (±0.11)	0.25 (±0.00)	No	No
	60% (60%)	0.83 (±0.23)	1.33 (±0.11)	No	No
	30% (30%)	0.75 (±0.00)	1.58 (±0.11)	No	No
Raspberry	80% (80%)	0.75 (±0.20)	0.58 (±0.11)	No	No
	60% (60%)	0.58 (±0.20)	0.58 (±0.11)	No	No
	30% (30%)	0.0 (±0.00)	0.0 (±0.00)	No	No
Thyme	80% (2%)	1.25 (±0.40)	0.83 (±011)	No	No
	60% (1.2%)	1.41 (±0.51)	0.66 (±0.11)	No	No
	30% (0.6%)	0.25 (±0.20)	0.0 (±0.00)	No	No
Field Horsetail	80% (2.5%)	0.0 (±0.00)	0.0 (±0.00)	No	No
	60% (1.5%)	0.16 (±0.11)	0.16 (±0.23)	No	No
	30% (0.75%)	0.66 (±0.23)	0.25 (±0.35)	No	No
Peppermint *	80% (3.5%)	0.0 ^cc^ (±0.00)	0.0 ^cc^ (±0.00)	No	No
	60% (2.1%)	1.92 ^cc^ (±0.23)	3.16 ^cc^ (±0.35)	No	No
	30% (1.05%)	4.41 ^d^ (±0.23)	4.17 ^d^ (±0.11)	Yes	Yes
Aloe vera	80% (80%)	0.75 (±0.20)	1.16 (±0.11)	No	No
	60% (60%)	1.08 (±0.62)	0.83 (±0.31)	No	No
	30% (30%)	0.83 (±0.11)	1.16 (±0.11)	No	No
Asiatic pennywort	80% (2%)	1.0 (±0.00)	0.0 (±0.00)	No	No
	60% (1.2%)	1.25 (±0.35)	0.0 (±0.00)	No	No
	30% (0.6%)	0.66 (±0.31)	0.0 (±0.00)	No	No
Lime	80% (2%)	0.50 (±0.35)	0.0 (±0.00)	No	No
	60% (1.2%)	0.08 (±0.11)	0.0 (±0.00)	No	No
	30% (0.6%)	0.66 (±0.11)	0.0 (±0.00)	No	No
Lemon balm	80% (2%)	1.91 (±0.23)	2.25 (±0.35)	No	No
	60% (1.2%)	1.5 (±0.35)	1.83 (±0.23)	No	No
	30% (0.6%)	1.33 (±0.23)	1.0 (±0.54)	No	No
Cucumber	80% (2%)	0.0 (±0.00)	0.0 (±0.00)	No	No
	60% (1.2%)	0.25 (±0.35)	0.25 (±0.35)	No	No
	30% (0.6%)	0.08 (±0.11)	0.08 (±0.11)	No	No
Common nettle	80% (3%)	0.0 ^d^ (±0.0)	0.0 ^d^ (±0.0)	No	No
	60% (1.8%)	0.25 (±0.20)	1.50 (±0.20)	No	No
	30% (0.9%)	1.83 (±0.42)	1.16 (±0.11)	No	No
Fenugreek *	80% (3%)	0.0 ^cc^ (±0.00)	0.0 ^cc^ (±0.00)	No	No
	60% (1.8%)	2.58 ^cc^ (±0.23)	2.4 ^cc^ (±0.11)	No	No
	30% (0.9%)	2.16 (±0.31)	1.08 (±0.11)	No	No

**—The difference was calculated between the control and the tested sample, *—Cytotoxic effect, BSA—low soiling level (bovine serum albumin 3.0 g/L), BSA + YE—high soiling level (bovine albumin 10 g/L+10 g/L); ^cc^—results are presented after applying cytotoxicity neutralization and reduced additionally by result of the medium’s antiviral activity; ^d^—results are presented after being additionally reduced by the result of medium antiviral activity.
